# Supplementary material for: Lower Energy Intake among Advanced vs. Early Parkinson’s Disease Patients and Healthy Controls in a Clinical Lunch Setting: A Cross-Sectional Study
Source: Nutrients. 2020 Jul 16;12(7):2109. doi: 10.3390/nu12072109 (PMC7400863; doi:10.3390/nu12072109)

Supplementary Material

Table S1. Additional regression models exploring explanatory variables (more than 10% effect on the primary outcome model) for the lower energy intake among advanced stage Parkinson disease patients in comparison to healthy controls (exploratory analysis).

Explanatory models	*B*	*t*	*p*	Lower Bound 95% Confidence Interval for B	Upper Bound 95% Confidence Interval for B
**Spoonfuls**					
Sex	183.045	4.004	0.000	91.493	274.598
Early PD	24.435	0.491	0.625	-75.256	124.126
Advanced PD	-99.466	-1.975	0.053	-200.296	1.364
Spoonfuls	9.259	5.867	0.000	6.098	12.419
**UE Tremor**					
Sex	310.753	5.833	0.000	204.063	417.443
Early PD	66.672	0.995	0.324	-67.541	200.885
Advanced PD	-127.330	-1.821	0.074	-267.331	12.672
Tremor UE	-21.665	-1.057	0.295	-62.692	19.362
**Eating problems**					
Sex	304.080	5.838	0.000	199.731	408.428
Early PD	43.816	0.701	0.486	-81.464	169.095
Advanced PD	-135.645	-2.094	0.041	-265.410	-5.879
Self-reported eating Problems	-58.564	-0.958	0.342	-181.021	63.894
**Dysphagia**					
Sex	301.607	5.809	0.000	197.629	405.585
Early PD	41.079	0.659	0.512	-83.683	165.841
Advanced PD	-139.809	-2.152	0.036	-269.886	-9.731
Dysphagia	-74.294	-1.092	0.279	-210.539	61.952

* = Model is significant, P<0.05. Sex: 1 = male, 0 = female. PD = Parkinson's disease. *B* = unstandardized *b* coefficients showing the change in kcal, *t* = the *t* test statistic, *p* = the probability value. UE = upper extremity.

Table S2. Additional regression models exploring variables that enhanced the lower energy intake more than 10% among advanced stage Parkinson disease patients in comparison to healthy controls (exploratory analysis).

Explanatory models	*B*	*t*	*p*	Lower Bound 95% Confidence Interval for B	Upper Bound 95% Confidence Interval for B
**Brady-/hypokinesia**					
Sex	297.363	5.686	0.000	192.632	402.095
Early PD	26.81	0.350	0.728	-124.965	177.927
Advanced PD	-182.928	-2.084	0.042	-358.678	-7.179
UE Brady-/hypokinesia	3.249	0.337	0.737	-16.038	22.535
**Water intake**					
Sex	299.814	5.766	0.000	195.692	403.936
Early PD	31.635	0.500	0.619	-94.967	158.238
Advanced PD	-186.555	-2.776	0.007	-321.144	-51.966
Water intake	-0.307	-0.931	0.356	-0.967	0.353
**Constipation**					
Sex	326.572	5.852	0.000	214.599	438.545
Early PD	59.796	0.903	0.370	-73.017	192.609
Advanced PD	-188.560	-2.772	0.008	-325.073	-52.046
Constipation	-10.381	-0.144	0.886	-154.874	134.111
**Smell problems**					
Sex	294.411	5.511	0.000	187.388	401.434
Early PD	7.384	0.085	0.933	-166.588	181.357
Advanced PD	-195.398	-2.261	0.028	-368.548	-22.247
Smell problems	49.313	0.586	0.560	-119.239	217.864

* = Model is significant, $P<0.05$. Sex: 1 = male, 0 = female. PD = Parkinson's Disease. *B* = unstandardized *b* coefficients, *t* = the *t* test statistic, *p* = the probability value. UE = upper extremity.

Table S3. Additional regression models exploring explanatory variables (more than 10% effect on the primary outcome model) for the lower energy intake among advanced stage Parkinson disease patients in comparison to early PD patients (exploratory analysis).

Explanatory models	*B*	*t*	*p*	Lower Bound 95% Confidence Interval for B	Upper Bound 95% Confidence Interval for B
**Spoonfuls**					
Sex	183.045	4.004	0.000	91.493	274.598
Healthy control	-24.435	-0.491	0.625	-124.126	75.256
Advanced PD	-123.901	-2.378	0.021	-228.226	-19.576
Spoonfuls	9.259	5.867	0.000	6.098	12.419
**Eating problems**					
Sex	304.080	5.838	0.000	199.731	408.428
Healthy control	-43.816	-0.701	0.486	-169.095	81.464
Advanced PD	-179.460	-2.751	0.008	-310.118	-48.803
Eating problems	-58.564	-0.958	0.342	-181.021	63.894
**Dysphagia**					
Sex	301.607	5.809	0.000	197.629	405.585
Healthy control	-41.079	-0.659	0.512	-165.841	83.683
Advanced PD	-180.888	-2.733	0.008	-313.415	-48.361
Dysphagia	-74.294	-1.092	0.279	-210.539	61.952

* = Model is significant, $P<0.05$. Sex: 1 = male, 0 = female. PD = Parkinson's Disease. *B* = unstandardized *b* coefficients showing the change in kcal, *t* = the *t* test statistic, *p* = the probability value.

Table S4. Additional regression models exploring variables that enhanced the lower energy intake more than 10% among advanced stage Parkinson disease patients in comparison to healthy controls (exploratory analysis).

Explanatory models	*B*	*t*	*p*	Lower Bound 95% Confidence Interval for B	Upper Bound 95% Confidence Interval for B
**Constipation**					
Sex	326.572	5.852	0.000	214.599	438.545
Healthy control	-59.796	-0.903	0.370	-192.609	73.017
Advanced PD	-248.356	-3.584	0.001	-387.399	-109.312
Constipation	-10.381	-0.144	0.886	-154.874	134.111

* = Model is significant, P<0.05. Sex: 1 = male, 0 = female. PD = Parkinson's Disease. *B* = unstandardized *b* coefficients showing the change in kcal, *t* = the *t* test statistic, *p* = the probability value.

Figure S1. Participant flowchart.

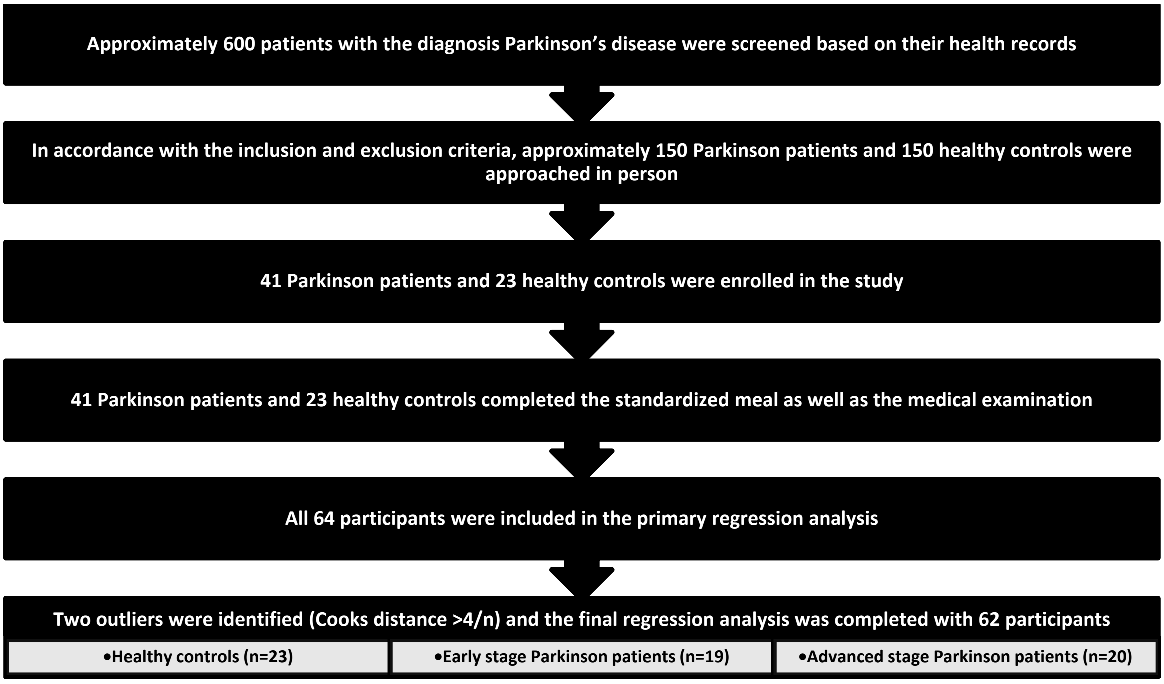

Supplement: Supplementary file 1 [file nutrients-12-02109-s001.pdf]
